# Stroke Burden in Agrı: Stroke Situation in Underdeveloped Region of Turkey

**DOI:** 10.5152/eurasianjmed.2021.20034

**Published:** 2021-10

**Authors:** Sezgin Kehaya

**Affiliations:** Department of Neurology, Trakya University School of Medicine, Edirne, Turkey

**Keywords:** Cerebral hemorrhage, stroke, atrial fibrillation, cardio embolism

## Abstract

**Objective:**

Stroke is the second cause of mortality and the third cause of disability worldwide in adults. There is no published data about stroke in Agrı. We aim to define stroke subtypes and associated risk factors. Thus, we can be aware of stroke burden in rural areas and can develop strategies to reduce the stroke risk.

**Materials and Methods:**

Records of Agrı State Hospital were investigated for a period of 3 years retrospectively. Patients were divided to ischemic and hemorrhagic stroke groups. Hemorrhagic strokes were classified as subarachnoid hemorrhage (SAH) and intracerebral hemorrhage (ICH). Ischemic strokes were classified according to localization and etiology. Vascular risk factors for ischemic stroke were assessed and compared between groups. The differences between variables were evaluated using Pearson κi square test and one-way ANOVA.

**Results:**

There were 1246 patients evaluated for stroke in Emergency Department and 525 (42% of pre-diagnosis) patients were diagnosed as stroke. There were 25.3% hemorrhagic (5.1% SAH and 20.1% ICH) versus 74.6% ischemic stroke. Intensive care required for 61.6% of hemorrhagic and 22.4% of ischemic patients. The most prevalent localization was partial anterior circulation infarction, and the most known etiology was cardioembolism after unknown cause in ischemic strokes. The most encountered risk factor was hypertension (HT). Coronary artery disease history, HT, and atrial fibrillation (AF) were risk factors for recurrent stroke (*P* = .001).

**Conclusion:**

Stroke types in Agrı resembles more to Asian population than Europe. Awareness for stroke, HT, and AF treatment could be the primary targets for stroke reduction in underdeveloped regions.

## Introduction

Stroke is defined as a sudden neurological deficit lasting more than 24 hours caused by hemorrhage or ischemia. After the new tissue based definition of transient ischemic attack, in which brain Magnetic Resonance imaging for proven ischemia was used, ischemic stroke definition could be made by sudden neurological deficit with proven infarction on imaging. Without including time concern. It is reported to be the second most common cause of mortality and the third cause of disability worldwide in adults.^1^ The prevalence of ischemia or hemorrhage differs among different geographic and ethnic populations. The European Registers of Stroke study reported 81.7% ischemia, 12.4% intracerebral hemorrhage (ICH) and 2.9% subarachnoid hemorrhage (SAH) across Europe.^2^ Old studies reported increased risk of hemorrhagic stroke and small vessel disease in Chinese population than whites, but a recent study reported only slight increase in hemorrhagic stroke and similar etiology in ischemic stroke nowadays.^3^ Epidemiologic stroke studies in Turkey are rare. Turkish Multicenter Stroke Study reported 29% hemorrhage and 71% ischemia in Turkish stroke patients.^4^ A single center study conducted in Adıyaman city of Turkey, reported 87.8% ischemic and 8.6% hemorrhagic stroke.^5^ A multicenter study for regional stroke risk difference in hypertensive patients at Turkey (THİNK trail), reported West Anatolia region to have 18% stroke risk for 10 years period.^6^ There is no any published data about stroke in Agrı, which is one of the least developed city of Turkey. There were 3 hospitals in Agrı city and Ağrı State Hospital was the main hospital, who givecare for stroke patients. Turkish Statistical Institute reported that 45.5% of population lives at rural areas of Agrı in 2014. That means nearly half of the population lives away from hospitals.

We aim to define stroke subtypes and associated risk factors in this study in Agrı. With these data we can be aware of stroke burden in rural areas and can develop strategies to reduce the stroke risk.

## Materials and Methods

After obtaining governmental permission from Turkish Public Hospitals Institution General Secretariat of Ağrı Province Public Hospitals Association with 10610145/755.02.06 numbered decision, we investigated electronic end printed records of Ağrı State Hospital Emergency and Neurology departments for 3 years, from October 19, 2013, to October 19, 2016, retrospectively. The study was approved by the Trakya University Non-interventional Studies Ethical Committee (TÜTF-BAEK 2017/120 no: 08/07). Because of retrospective nature of the study no informed consent of the patients have been obtained.

All ICD codes for ischemic, hemorrhagic stroke and transient ischemic attack were investigated from electronic VETA system coded by Emergency or Neurology departments. Patients over 18 years old were included to the study. Stroke within 7 days of symptom was defined as acute stroke and included to the study. Patients with head trauma and transient ischemic attack were excluded. Computerized tomography (CT), magnetic resonance imaging’s (MRI), and neurology consultation notes of all patients were investigated. All ischemic stroke patient imagings with CT or MRI were investigated and lesions which explained the clinical situation were defined as stroke. If consultation notes were not in the electronic system, printed files of the patients were investigated.

Patients were divided to ischemic and hemorrhagic stroke groups as defined in previous studies.^2,3^ Hemorrhagic strokes were classified as ICH and SAH. Hypertension (HT) status and medication use were noted. Known HT was defined as previous anti-hypertensive medication use and presence of HT was defined as tension over 140/90 mm Hg on repeated measurements after 1 week of stroke.^2^ ICH were classified as hypertensive hematoma and lobar hematoma. The localization of hypertensive hematoma was noted as thalamic, basal ganglionic, brain stem and cerebellar. The relation between hemorrhage type and intensive care need was investigated.

Ischemic stroke patients, who were investigated in Ağrı State Hospital neurology service, were classified according to localization and etiology. Patients with transient ischemic attacks were excluded and only definitive stroke patients were investigated. Bamford’s criteria were used for localization as total anterior circulation infarction (TACI), partial anterior circulation infarction (PACI), posterior circulation infarction (POCI), and lacunar infarction (LACI).^7^ For etiologic classification Trial of Org 10,172 in acute ischemic stroke (TOAST) criteria were used as cardio embolism (CE), large artery atherosclerosis (LAA), small vessel disease (SVD), unknown cause (UC), and other known cause (OC).^8^ For CE evaluation, echocardiography, 12 lead electrocardiography and 24 hour Holter electrocardiography results were investigated. For LAA, MR angiography, CT angiography, or carotid Doppler ultrasonography findings were used and occlusion or stenosis of arterial wall over 50% were used for diagnosis. If we cannot obtain the imaging recordings or both CE and LAA diagnosis criteria were present, the case was diagnosed as UC.

Following previous studies, traditional vascular risk factors for ischemic stroke were assessed.^2,5,9^ The risk factors evaluated were arterial HT (defined as antihypertensive use or repeated measures >140/90 mm Hg), hyperlipidemia (HL) (defined as medication use or fasting serum total cholesterol of >200 mg/dL, low density lipoprotein cholesterol >160 mg/dL, triglyceride >250 mg/dL), diabetes mellitus (DM) (defined as medication use or Hgb A1c >6.5, fasting glucose >125 mg/dL, 2 measures of postprandial serum glucose over >200 mg/dL), atrial fibrillation (AF) (AF at electrocardiography or paroxysmal AF at 24 hour Holter monitoring), coronary artery disease (CAD) history and previous stroke history.

Statistical analyses were performed using SPSS 20 version for Windows (IBM SPSS Corp.; Armonk, NY, USA). The frequencies of variables were evaluated as noun and percent. The differences between categorical variables were evaluated using Pearson κi square or Fisher exact test. The differences between continuous variables were evaluated using the Student *t* test, the Mann–Whitney *U*-test and one way ANOVA where appropriate. The value of *P* < .05 was considered statistically significant.

## Results

Between October 19, 2013, and October 19, 2016, there were 1630 pre-diagnosis as cerebrovascular diseases at Emergency recordings. After exclusion of repeated patient records and patients referred after head trauma 1246 patients were evaluated for stroke. When radiological findings and consultation notes were investigated, 525 (42.13% of pre-diagnosis) patients were diagnosed as stroke (including transient ischemic attacks) by neurology specialist. Only 3 patients referred to emergency in 3 hours period of symptom onset, which was thrombolysis period that time. Because of unavailability none of these patients could receive thrombolysis treatment. There were no thrombolytic agent in the hospital and the closest hospital, who gives the treatment, were 2 hours away. There were 3 hospitals in Agrı, 2 had MRI machines and the other hospital had no neurologist in half time at the study period. That was why these patients could not receive proper treatment.

In all, 133 (25.33%) patients were hemorrhagic stroke (5.14% SAH and 20.19% ICH) versus 392 (74.67%) ischemic stroke. According to the hospitalization status of these patients 54 (40.6%) of 133 hemorrhagic patients were transferred for intensive care to another hospital and 79 (59.4%) patients hospitalized at Ağrı State Hospital [28 (21.05%) at intensive care unit and 51 (38.34%) at neurology service]. In another word, 82 (61.65%) of 133 hemorrhagic patients needed intensive care. Fifty three (13.52%) of 392 ischemic patients were transferred for intensive care to another hospital and 339 (86.48%) patients hospitalized at Ağrı State Hospital [35 (8.93%) at intensive care unit and 304 (77.55%) at neurology service]. From 392 ischemic patients, 88 (22.45%) patients needed intensive care.

Additionally to Emergency recordings, we investigated Neurology service recordings. Because there were acute stroke patients hospitalized directly from out-patient neurology clinic and directly received to Neurology service from other 2 hospitals in Agrı. There were 462 in-patients at neurology service diagnosed as stroke. Sixty six (14.29%) patients were hemorrhagic stroke and 396 (85.71%) patients were ischemic stroke. After reviewing all files of these patients from emergency and service together, we obtained 146 hemorrhagic and 408 ischemic stroke patients.

Table 1 shows demographics of hemorrhagic strokes. Basal ganglionic hematoma (36.3%) was the most prevalent hemorrhagic stroke type followed by SAH (18.5%). Female gender was none significantly more prevalent in SAH and male were more prevalent in other groups. There was no association between gender and HT status (73.6% of males and 72.9% of females had HT). New HT diagnosis was made mostly in HT hematoma group. Intensive care need was mostly encountered for SAH, followed by BG hematoma and lobar hematoma. Intensive care need was not related with gender or age. When we looked for HT status and intensive care need, we found that 63.4% of patients followed in intensive care unit and 85.9% of patient followed in neurology service had HT. That means HT was inversely associated with intensive care need (*P* = .002). The patients who had not HT and needed intensive care were mostly in SAH group.

Tables 2 and 3 show demographic features of risk factors according to localization and etiology in ischemic stroke patients. The most prevalent localization was PACI, and TACI patients were older than other localizations. AF was more prevalent in TACI group than others (*P* < .001). Post hoc Tukey analyses showed AF was more prevalent in TACI then POCI and LACI (*P* < .001), but not from PACI (*P* = .215). Males with AF had more TACI than females (43.2% vs 31% of TACI). There were no statistically significant association between localization classification and presence of vascular risk factors except AF. According to etiology, UC was the most prevalent group followed by CE. Post hoc analyses showed that patients with CE were statistically older than OC, UC, and SVD but not from LAA. OC (all were hematological tendency for thrombosis) were younger than other groups (*P* < .001). OC patients had no traditional vascular risk factors and because 0 count in this group our statistics between etiology and risk factors could be effected. When we done our statistics, excluding OC group, the significance between risk factors and etiologic groups persisted. CE patients had more CAD and previous stroke history. At subgroup analyses for gender, stroke history was correlated with CE in male (*P* = .028) but not in female (*P* = .456). PACI was the most frequent localization in all etiologic groups. LAA was more prevalent in males and had more dyslipidemia and DM. SVD had statistically more HT, DM, dyslipidemia as expected. There was significant difference between etiology and localization. LAA and CE were presented more as PACI and TACI then others. UC was more encountered as PACI and least as TACI.

**Figure 1.**
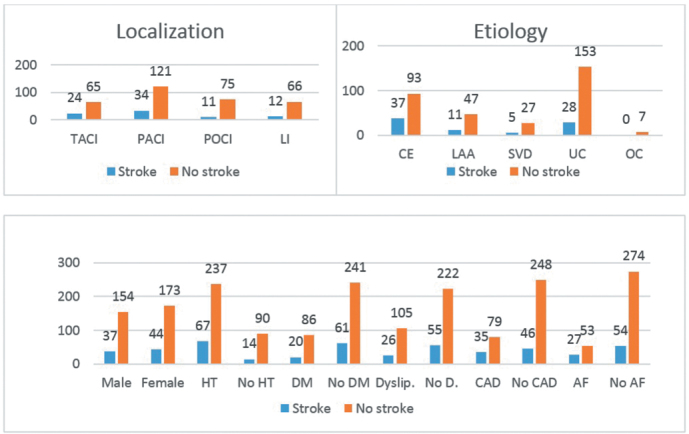
Relationship between localization, etiology, vascular risk factors, and previous stroke history in ischemic stroke

We compared localization, etiology, and vascular risk factors according to having a previous stroke. Figure 1 demonstrates the number of patients’ according risk factors. TACI (27%) had the most frequent previous stroke history followed by PACI (21.9%) (*P* = .074). CE (28.5%) had more previous stroke history than LAA (19%), SVD (15.6%), and UC (15.5%) (*P* = .035). There were no association between gender, DM, dyslipidemia and stroke history. Patients with HT had a more previous stroke then patients without HT (22% vs 13.5%, *P* = .058). CAD history (30.7% vs 15.6%) and AF (33.8% vs 16.5%) were statistically more frequent in previous stroke history (*P* = .001).

## Discussion

We observed more hemorrhagic strokes then European and less then Asian populations. Thalamic and basal ganglionic localizations were most prevalent regions of hemorrhage in ICH. HT was the most prevalent risk factor for hemorrhagic stroke. Among ischemic strokes PACI was the most frequent localization and CE the most frequent etiology after UC. The most prevalent risk factor for ischemic stroke was HT followed by dyslipidemia and CAD. We observed higher percent of intensive care need for stroke then Europe. HT, CAD history and AF were risk factors for recurrent stroke. With higher hemorrhagic stroke rate and ischemic stroke subtype profile we can state that Agrı city stroke burden was between Europe and Asia but resembles more to the Asian population than European.

Only 3 patient referred to the emergency at the time of thrombolysis period in our study. There were acute stroke patients referred to out-patient neurology clinic. Stroke diagnosis accuracy by emergency specialists or general practitioners, who were care givers at Emergency department, was only 42%, which was a low number too. That means stroke awareness was very low in Agrı.

In our study, we observed 5.1% SAH, 20.2% ICH and 74.7% ischemic stroke in Agrı. Stroke prevalence and stroke types can vary according to geographical, ethnic and socioeconomic factors.^2,3,10^ A multicenter study in Europe reported 81.7% ischemic, 2.9% SAH and 12.4% ICH (2). Chinese population was reported to have more hemorrhagic stroke then whites and an epidemiologic study which compared white versus Chinese population in China and Taiwan reported ICH prevalence of 33% vs 12% in favor of Chinese.^3^ But another epidemiological Chinese study observed transition in stroke rates in time.^11^ They reported that, incidence rate of hemorrhagic stroke declined by 1.7% and ischemic stroke increased by 8.7%. In previous studies conducted at Turkey, Özdemir et al.^4^ reported 71% ischemic and 29% hemorrhagic stroke across Turkey in 2000. In single center data of two cities socioeconomically more developed than Agrı, Adıyaman University Hospital reported 87.8% ischemia and 8.6% hemorrhage; Ege University Hospital reported 77% ischemic stroke, 19% ICH and 4% SAH.^5,12^ Ischemic and hemorrhagic stroke proportions in nearby Countries of Turkey reported 80.5–86.6% ischemia versus 13.4–19.6% hemorrhage in Greece; and 69–78% ischemic versus 22–31% hemorrhage in Pakistan.^13,14^

In our study, 61.65% of hemorrhagic strokes and 22.45% of ischemic strokes needed intensive care. This high percent of intensive care need of hemorrhagic strokes was due to SAH, of which 92.6% needed intensive care. It is reasonable because the main etiology of SAH was aneurismal rupture and surgery, as well as postoperative intensive care was needed. A German cohort study reported 374 (7.5%) intensive care need of 4958 consecutive stroke patients referred to stroke unit.^15^ This large difference maybe because there was no stroke unit in Ağrı State Hospital and that study did not included the SAH patients. We found an inverse association with intensive care need and presence of HT, which was mainly related to high percent of non-hypertensive SAH patients.

The Global Burden of Disease 2010 Study showed 47% increase in the absolute number of hemorrhagic stroke cases worldwide since 1990, while reduced by 8% in high-income countries.^16^ This transition was correlated with HT control programs. The Global Burden of Disease reported age specific trends in 2013 and 2016, but we preferred 2010 data because of hemorrhagic stroke data was showed only there. The most prevalent risk factor for ICH was HT followed by increased age, Asian race and alcohol intake. The cause of lobar hematoma was known to be mainly cerebral amyloid angiopathy, vascular malformations, drugs, hemorrhagic diathesis and HT, too. Deep hematomas were mainly due to hypertensive vasculopathy.^17^ The risk factors for ischemic and intracerebral haemorrhagic stroke in 22 countries (INTERSTROKE) study, which was a case control study, reported HT as the most prevalent risk factor.^9^ They observed HT in 83% of ICH and 66% of ischemic strokes. The localization patterns of ICH in our study, with high BG and thalamic localization, was like the study for in hospital mortality risk factors in hemorrhagic strokes, done in Tehran/Iran, which is a neighbor city to Agrı.^18^ Arboix et al.^19^ investigated early outcome after primary ICH in Spain and reported lobar hematoma as the most prevalent localization of hemorrhage in their study. According to these data, Agrı resembles more to eastern countries than Europe in sense of hemorrhagic stroke prevalence and localization.

The most prevalent localization of ischemia in our study was PACI similar to INTERSTROKE study, which was done in multi-continental nature, but we observed more TACI and POCI compared to them.^9^ We also observed more CE and less SVD. Our study had 44.4% UC, but these studies had UC between 22% to 52%, too. Previous studies in Turkey showed 27% CE in Aegean region.^12^ Increased age was a risk factor for TACI localization and CE etiology. Increased risk of CE by advanced age was reported previously, and this was consistent with increased AF prevalence with aging.^20^ The most prevalent risk factor for ischemic stroke in our study was HT followed by dyslipidemia, CAD and DM. HT is the most prevalent vascular risk factor for stroke and it is showed that with treatment of HT, stroke risk could be reduced 22%.^21^ DM is another prevalent risk factor for stroke but with glycemic control, there were no reduction of macro vascular complications like stroke. Two large studies, ACCORD and ADVENCE, investigated relation between glycemic control and vascular complications.^22^ The ACCORD trail was stopped earlier because of high mortality in tight glycemic control and ADVANCE showed no reduction in stroke risk. The THİNK trail reported 29% DM and 22% CAD history in hypertensive patients in Turkey.^6^ In our study similarly, 30.9% of our ischemic patients with HT, had DM (not showed in results). This could be an opportunity, that with proper HT treatment strategies, stroke could be reduced in a high percent in Agrı. Our study observed 32.1% dyslipidemia in ischemic strokes like previous study in Adıyaman/Turkey. A meta-analysis observed 24% risk reduction for stroke with each 39-mg/dL lowering of LDL-C by statins.^23^ In another word, one third of our ischemic patients could benefit from lipid lowering therapy. We observed AF in 19.6% of our ischemic patients and 32.6% of TACI, which in the most severe stroke causing disability. The TEKHARF study reported AF prevalence in 1.25% of general population and 2.49% in people over 70 years in Turkey.^24^ The AFTER study observed that only 42% of high risk patients in Turkey received anticoagulant therapy.^25^ AHA/ASA 2014 Guideline for primary prevention of stroke reported 4- to 5-fold increased risk of ischemic stroke with AF and advised pulse measurement followed by Electrocardiography in every patient over 65 years.^26^ With increasing awareness of AF many strokes could be prevented in Agrı. We observed CE as the most prevalent recurrent stroke etiology. CAD and AF were statistically related with stroke recurrence and HT was nearly statistically significant, too. The relation between CAD and stroke was reasonable because both had the same vascular risk factors. Previously AF, CAD, HT and DM ware correlated with recurrent stroke.^26,27^ AF and HT were proposed as potential targets to reduce recurrence.^27^

The retrospective design and short period of time ware the most important limitations of this study. Short time and single hospital based data of our study could not give us epidemiologic data as incidence and prevalence of stroke types in Agrı city. We obtained data from recordings that’s why we could not give data about risk factors that were not recorded like smoking, diet, physical activity, obesity, and alcohol intake. This could affect the risk factor burden and create bias among risk factor profiles. Half of the population in Agrı lived in rural areas and there were 3 hospitals in Agrı city. The socio-economic status in Agrı was very low. We had stoke patient internated form out-patient clinic in 3rd day of stroke. There are 2 big cities near Agrı, Van and Erzurum, which could give care for stroke. Obviously our data cannot show the exact stroke burden in all Agrı city and could had selection bias. But Ağrı State Hospital was the main hospital, who was given stroke care and the other hospitals had no neurologist in half time at the study period. And the other 2 big cities were at least 2 hours away from the center. This made Ağrı State Hospital the most referred hospital for stroke. That’s why we believe that our data represent the stroke burden in Agrı although it was single center and hospital based one. We think our results could be repeated and generalized for Agrı city.

Despite the previously mentioned limitations of this study we think that our data is valuable because there was no any data about stroke burden in underdeveloped regions of Turkey. In scope of our findings we can state that the ischemic and hemorrhagic burden of stroke in Agrı resembles much more to Asian population than Europe. We observed 25.3% hemorrhagic stroke and CE etiology was more profound than LAA as Asian countries. This can be interpreted as patients in Agrı city may have more hemorrhagic stroke etiology or have increased risk of hemorrhagic transformation in ischemic strokes due to CE etiology, than western part of Turkey. Uncontrolled HT and AF were statistically significant risk factors for stroke. These chronic risk factors could be controlled with routine follow-up, which means awareness of the risk of stroke with these risk factors plays the most important role.

As a conclusion to decrease stroke risk and improve outcomes of stroke, awareness for stroke and its risk factors seems to be the primary target for Agrı. HT in hemorrhagic strokes; HT and AF in ischemic stokes appear to be the most abundant vascular risk factors to target for stroke reduction. But still multicenter prospective studies are needed.

**Main Points**

- Stroke burden in Agrı, which is an underdeveloped city in eastern region of TURKEY, resembles much more to Asian population than Europe in terms of high hemorrhagic stroke rate and high cardioembolic isceamic stroke etiology.
- The awareness of stroke was low among general practitioners and the folk.
- Uncontrolled HT was the most prevalent risk factor for stroke. HT, CAD and AF were prevalent risk factors for requrrent stroke which could be changed.

## Figures and Tables

**Table 1.** Hemorrhagic Stroke Demographics

Hemorrhage Type	SAH (n = 27)	Hypertensive Hematoma (n = 95)					Lobar Hematoma (n = 24)	*P*
		BG (n = 53)	Thalamic (n = 26)	Brainstem (n = 3)	Cerebellar (n = 13)	Total (n = 95)		
Patient %	18.5	36.3	17.8	2.1	8.9	65.1	16.4	
Male %	48.1	60.4	57.7	66.7	76.9	62.1	62.5	.40
Mean age (years ± SD)	62 ± 12	66 ± 11	65 ± 12	54 ± 24	65 ± 19	65 ± 13	66 ± 13	.53
Presence of HT %	40.7	81.1	88.5	66.7	84.6	83.2	70.8	<.001
Known HT %	40.7	60.4	69.2	66.7	76.9	65.3	66.7	<.001
Intensive care need %	92.6	58.5	34.6	33.3	38.5	48.4	45.8	<.001

BG: basal ganglia, HT: arterial hypertension, p: association between SAH, hypertensive hematoma, and lobar hematoma, SAH: sub arachnoid hemorrhage

**Table 2.** Demographic Findings of Ischemic Strokes according to Localization

	TACI (n = 89, 21.8%)	PACI (n = 155 38%)	POCI (n = 86 21.1%)	LACI (n = 75 19.1%)	Total (n = 408 100%)	*P*
Age (years)	74.1 ± 13	72.8 ± 12	66.3 ± 15	65.2 ± 12	70.3 ± 13	<.001
Male (n/%)	42/47.2	65/41.9	46/53.5	38/48.7	191/46.8	.371
HT (n/%)	70/78.7	112/72.3	61/70.9	61/78.2	304/74.5	.501
DM (n/%)	27/30.3	39/25.2	23/26.7	17/21.8	106/26	.644
CAD (n/%)	26/29.2	43/27.7	27/31.4	18/23.1	114/27.9	.682
Stroke (n/%)	24/27	34/21.9	11/12.8	12/15.4	81/19.9	.074
Dyslipidemia (n/%)	27/30.3	46/29.7	31/36	27/34.6	131/32.1	.709
AF (n/%)	29/32.6	35/22.6	9/9.3	8/10.3	80/19.6	<.001

According to Bamford’s criteria, AF, atrial fibrillation; CAD, coronary artery disease; DM, diabetes mellitus; HT, hypertension; LACI, lacunar infarction; PACI, partial anterior circulation infarction; POCI, posterior circulation infarction; TACI, total anterior circulation infarction.

**Table 3.** Demographic Findings of Ischemic Strokes according to Etiology

	CE (n = 130, 31.9%)	LAA (n = 58, 14.2%)	SVD (n = 32, 7.8%)	UC (n = 181, 44.4%)	OC (n = 7, 1.7%)	Total (n = 408, 100%)	*P*
Age (years)	74.6 ± 11	70.6 ± 14	63.8 ± 9	69.6 ± 13	32.8 ± 14	70.3 ± 13	<.001
Male (n/%)	64/49.2	32/55.2	18/52.2	77/42.5	0	191/46.8	.032
HT (n/%)	97/74.6	43/74.1	30/93.8	134/74	0	304/74.5	<.001
DM (n/%)	32/24.6	20/34.5	18/56.2	36/19.9	0	106/26	<.001
CAD (n/%)	66/50.8	13/22.4	7/21.9	28/15.5	0	114/27.9	<.001
Stroke (n/%)	37/28.5	11/19	5/15.6	28/15.5	0	81/19.9	.035
Dyslipidemia (n/%)	33/25.4	18/31	20/62.5	60/33.1	0	131/32.1	.001
AF (n/%)	77/59.2	0	0	3/1.7	0	80/19.6	<.001
TACI (n/%)	46/35.4	18/31	0	25/13.8	0	89/21.8	<.001
PACI (n/%)	53/40.8	28/48.3	0	70/38.7	4/57.1	155/38	
POCI (n/%)	22/16.9	8/13.8	9/28.1	44/24.3	3/42.9	86/21.1	
LACI (n/%)	9/6.9	4/6.9	23/71.9	42/23.2	0	78/19.1	

According to TOAST etiologic classification, CAD: coronary artery disease, CE: cardio embolism, DM: diabetes mellitus, HT: hypertension, LACI: lacunar infarction, LLA: large artery atherosclerosis, OC: other known cause, PACI: partial anterior circulation infarction, POCI: posterior circulation infarction, SVD: small vessel disease, TACI: total anterior circulation infarction, UC: unknown cause
